# Increased Risk of Refractive Errors and Amblyopia among Children with Ptosis: A Nationwide Population-Based Study

**DOI:** 10.3390/jcm11092334

**Published:** 2022-04-22

**Authors:** Ning-Yi Hsia, Li-Yen Wen, Ching-Ying Chou, Cheng-Li Lin, Lei Wan, Hui-Ju Lin

**Affiliations:** 1 Department of Ophthalmology, China Medical University Hospital, Taichung 40402, Taiwan; deepwhite1111@hotmail.com; 2 School of Medicine, China Medical University, Taichung 40402, Taiwan; 3 School of Chinese Medicine, China Medical University, Taichung 40402, Taiwan; liyenwen0627@gmail.com (L.-Y.W.); u102022007@cmu.edu.tw (C.-Y.C.); 4 Management Office for Health Data, China Medical University Hospital, Taichung 40402, Taiwan; orangechengli@gmail.com; 5 Department of Medical Laboratory Science and Biotechnology, Asia University, Taichung 41354, Taiwan; 6 Department of Obstetrics and Gynecology, China Medical University Hospital, Taichung 40402, Taiwan

**Keywords:** ptosis, astigmatism, myopia, hyperopia, amblyopia

## Abstract

Background: This study aimed to investigate the risk of refractive errors (astigmatism, myopia, and hyperopia) and amblyopia in children with ptosis and association between age at diagnosis of ptosis and subsequent risks of vision problems. Methods: Retrospective claims data from the Taiwan National Health Insurance Research Database (NHIRD) were analyzed. We identified 1799 children aged 0–18 years who were newly diagnosed with ptosis between 2000 and 2012 and 7187 individuals without the disease. Both cohorts were followed up until 2013 to estimate the incidence of refractive errors and amblyopia. Results: Children with ptosis had 5.93-fold, 3.46-fold, 7.60-fold, and 13.45-fold increases in the risk of developing astigmatism, myopia, hyperopia, and amblyopia, respectively, compared with the control cohort (astigmatism: adjusted hazard ratio, aHR = 5.93, 95% confidence interval, CI = 5.16–6.82; myopia: aHR = 3.46, 95% CI = 3.13–3.83; hyperopia: aHR = 7.60, 95% CI = 5.99–9.63; amblyopia: aHR = 13.45, 95% CI = 10.60–17.05). Children diagnosed with ptosis at an age older than 3 years old had a higher risk of myopia than patients diagnosed with ptosis before age 3. There was no significant difference of the risk of astigmatism, amblyopia, and hyperopia between age groups. Conclusions: Children with ptosis may exhibit a higher risk of astigmatism, myopia, hyperopia, and amblyopia than children without ptosis. The risk of myopia is higher in children with ptosis diagnosed at >3 years than those diagnosed at ≤3 years.

## 1. Introduction

Ptosis refers to either unilateral or bilateral upper eyelids falling into a position that are lower than a normal level and contributes to the narrowing of the vertical palpebral fissure [1]. This disorder results from dysfunction of the muscles that control lid retraction, including levator palpebrae superioris (LPS), LPS aponeurosis, and Müller’s muscle, or the nerve innervates of levator muscle, such as the superior branch of oculomotor nerve and cervical sympathetic system [2,3]. Based on age of onset, ptosis can be classified as congenital or acquired [4]. The most common type of ptosis in childhood is congenital ptosis, which presents at birth or by 1 year of age [5]. In a 40-year period retrospective cohort study, the prevalence of childhood ptosis was 7.9 per 100,000 patients (younger than 19 years), and the congenital type comprised 76% of pediatric ptosis [6]. Even though the classic presentation of pediatric ptosis is an isolated and non-progressive condition, numerous studies have indicated that childhood ptosis was associated with abnormal visual development due to stimulus deprivation and pressure on the cornea [7,8,9].

Over the past decades, considerable attention has been paid to refractive errors and amblyopia in childhood. Refractive errors are the most common cause of visual impairment worldwide [10]. Emmetropia is a state of refraction that incidence of parallel rays of light from distant objects is brought to a focus upon the retina without accommodation. During postnatal eye growth, the precise matching of the axial length (the distance from the anterior corneal surface to the retina along the visual axis) and the optical power of the eye in order to achieve emmetropia is known as emmetropization [11,12,13]. Any disruption of emmetropization results in the development of refractive errors, wherein visual images are focused either behind (hyperopia) or in front (myopia) of the retina [11,12]. Astigmatism is also a type of refractive error caused by rotational asymmetry of refractive power along different meridians in the eye [14]. Amblyopia defines as a reduction in best-corrected visual acuity that cannot be attributed the cause of any structural abnormality of the eye or visual pathways [15]. The estimated prevalence of amblyopia is 1–5%, which is the leading cause of monocular vision impairment in children [16]. The abnormal binocular visual experience in childhood, such as anisometropia, refractive errors, and stimulus deprivation, may cause abnormal development of the visual cortex and result in amblyopia [15].

Numerous studies have investigated the relationship between amblyopia, refractive errors, and ptosis in childhood. It has been reported that the prevalence of refractive errors and amblyopia among children with ptosis is higher than in the general population [9,17,18]. However, most of the previous studies on pediatric ptosis focused on congenital ptosis, which means the diagnosis was made within a year after birth. There are limited studies on the impact of pediatric ptosis which developed after infancy. Thus, we used a nationwide database to investigate the risk of astigmatism, myopia, hyperopia, and amblyopia in ptosis children and further compared the risk regarding the age at diagnosis of ptosis.

## 2. Materials and Methods

### 2.1. Data Resource

Taiwan’s National Health Insurance (NHI) program began in March 1995 and includes information on up to 99% of the 23.74 million people living in Taiwan.

For this study, we used data files of children (aged < 18 years) from the National Health Insurance Research Database (NHIRD), which was established and is maintained by the National Health Research Institutes (NHRI). The dataset consisted of a random and nationally representative sample of half of all children in Taiwan who were insured from 2000 to 2013. All diagnoses and disease definitions were recorded using the International Classification of Diseases, Ninth Revision, Clinical Modification (ICD-9-CM) codes.

The study was approved by the Research Ethics Committee of China Medical University and Hospital in Taiwan (CMUH-104-REC2-115).

### 2.2. Sampled Subjects

Children younger than 18 years of age with ptosis (ICD-9-CM codes 374.30) were included in the ptosis cohort. For each child with ptosis, four children without an existing diagnosis of ptosis were randomly selected for the non-ptosis cohort with a frequency matching method to ensure both cohorts had the same distributions for strata of sex, age (every 1-year span), urbanization level, parental occupation, and index year of ptosis.

Children with a pre-existing diagnosis of any one of these four diseases: astigmatism (ICD-9-CM codes 367.2), myopia (ICD-9-CM codes 367.1), hyperopia (ICD-9-CM codes 367.0), and amblyopia (ICD-9-CM codes 368.00) were excluded.

### 2.3. Outcome Measures

The ptosis and non-ptosis cohorts were followed up until refractive errors (astigmatism, myopia, and hyperopia) or amblyopia occurred or were censored from the study because of failure to follow up (including withdrawal of insurance, immigration, and prison sentence), death, or the end of 2013.

The sociodemographic variables in this study were age, sex, urbanization level, and parental occupation (white collar, blue collar, or other). Based on population density (people per km^2^), the NHRI stratified all city districts and townships in Taiwan (based on national administrative zones demarcation) into several urbanization levels. Level 1 represents the most urbanized group, and level 4 indicates the least urbanized.

White collar workers were employees characterized by indoor work, including public institutional workers, educators, and administrative personnel in business and industries. Blue collar workers were characterized by increased hours of outdoor work, such as fishermen, farmers, and industrial laborers. Other occupations included mainly retired, unemployed, and low-income populations.

### 2.4. Statistical Analysis

We compared sociodemographic factors between the ptosis and non-ptosis cohorts using the Chi-square test. Student’s t test was used for continuous variables. The overall gender- and age-specific incidence densities (per 1000 person-years) were calculated by the number of events (astigmatism, myopia, hyperopia, and amblyopia) during the follow-up period divided by the total population at risk during the entire study period. Univariate and multivariate Cox proportional hazards regression models were used to estimate the hazard ratios (HRs) and 95% confidence intervals (CIs) for refractive errors and amblyopia, with stratification based on gender and age. The multivariable models were simultaneously adjusted for age, sex, urbanization level and parental occupation. We used the Kaplan–Meier method to calculate the cumulative incidence of refractive errors and amblyopia. The survival curves of the ptosis and non-ptosis cohorts were compared using the log-rank test. A p value less than 0.05 was considered statistically significant. We used SAS statistical package (version 9.4) to analyze all datasets.

## 3. Results

There were no differences in the distributions of sociodemographic characteristics between the ptosis and control groups (Table 1). The mean follow-up periods in the ptosis group were 5.20 (±3.62, standard deviation [SD]), 5.07 (±3.26, SD), 6.04 (±3.71, SD), and 5.75 (±3.71, SD) years for astigmatism, myopia, hyperopia, and amblyopia, respectively. As to the comparison groups, the mean follow-up times were 6.40 (±3.64, SD), 6.12 (±3.55, SD), 6.55 (±3.66, SD), and 6.58 (±3.65, SD) years for astigmatism, myopia, hyperopia, and amblyopia years, respectively.

The crude and adjusted HRs of risk factors for refractive errors (astigmatism, myopia, and hyperopia) and amblyopia revealed that the children who were diagnosed as having ptosis had a higher risk of refractive errors and amblyopia (astigmatism: aHR = 5.93, 95% CI = 5.16–6.82; myopia: aHR = 3.46, 95% CI = 3.13–3.83; hyperopia: aHR = 7.60, 95% CI = 5.99–9.63; amblyopia: aHR = 13.45, 95% CI = 10.60–17.05) (Table 2).

The incidence rates and HRs of refractive errors and amblyopia for both cohorts stratified by sex revealed that both boys and girls with ptosis had a higher risk of refractive errors and amblyopia (Table 3). In the boys group, the adjusted HRs for refractive errors and amblyopia were 6.36 (95% CI, 5.12–7.89), 3.22 (95% CI, 2.74–3.78), 7.69 (95% CI, 5.29–11.16), and 11.11 (95% CI, 7.79–15.84) for astigmatism, myopia, hyperopia, and amblyopia, sequentially. In the girls group, the adjusted HRs for astigmatism, myopia, hyperopia, and amblyopia were 5.67 (95% CI, 4.72–6.81), 3.63 (95% CI, 3.19–4.14), 7.53 (95% CI, 5.54–10.24), and 15.59 (95% CI, 11.31–21.51), respectively.

Children who were diagnosed as having ptosis after age of 3 had a higher risk of myopia than those before age of three. (Table 4). In the age ≤ 3 group, adjusted HRs were 6.08 (95% CI, 5.15–7.17), 3.14 (95% CI, 2.75–3.59), 7.03 (95% CI, 5.37–9.22), and 13.75 (95% CI, 10.46–18.07) for astigmatism, myopia, hyperopia, and amblyopia, sequentially. In the age >3 group, adjusted HRs for astigmatism, myopia, hyperopia, and amblyopia were 5.76 (95% CI, 4.46–7.45), 3.90 (95% CI, 3.34–4.56), 10.11 (95% CI, 6.15–16.62), and 13.21 (95% CI, 8.17–21.37), respectively.

Children in both cohorts who were diagnosed with ptosis from 2002 to 2012 had a significantly higher prevalence of amblyopia (Table 5).

**Figure 1:**
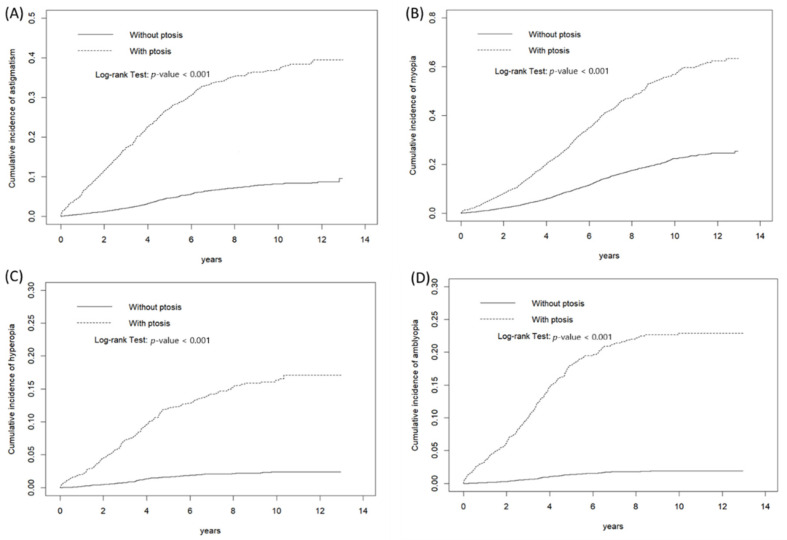
Cumulative incidence of (**A**) astigmatism, (**B**) myopia, (**C**) hyperopia, and (**D**) amblyopia in patients with and without ptosis.

Figure 1 shows that the cumulative incidences of (A) astigmatism, (B) myopia, (C) hyperopia, and (D) amblyopia of patients with and without ptosis differed significantly (log-rank test: *p* < 0.001).

## 4. Discussion

This nation-wide population-based study showed that children with ptosis had significantly increased risks of subsequently developing astigmatism, myopia, hyperopia, and amblyopia compared to children without ptosis. Moreover, children with ptosis diagnosed after the age of 3 exhibited a higher risk of myopia than patient diagnosed with ptosis under 3 years old. There is no significant difference in the risk of astigmatism, amblyopia, and hyperopia between early and late diagnosis age groups.

Several studies focused on the association between ptosis and myopia have been conducted. In animal models, the ocular changes observed in form-deprived eyes clearly demonstrated that degrading retinal image quality can produce robust myopic changes [19,20]. During the normal emmetropization process, visual deprivation causes increased eyeball growth and excessive increase in axial length [21,22,23]. In addition, we found that children with ptosis occurred or were diagnosed over age of 3 had a higher risk of myopia than children diagnosed at an age less than 3. Our result was consistent with previous scientific evidence, which indicated that prolonged ptosis leads to myopia by impairing the formation of a clear retinal image, and the prevalence of myopia increases with growing age [24]. This proposes the importance of the early treatment of ptosis and that the follow-up examination cannot be omitted or delayed.

The risk of hyperopia also increased among children with ptosis compared to the general population, which seems reverse to the results of myopia. Hyperopia produced in animals has been reported [25,26], and there may be different mechanisms then just an open-loop condition and reduced retinal image contrast or mid-range spatial frequency vision. In a recent paper by Zeng [27], the axial length (AL)/corneal curvature radius (CR) ratios of the ptotic eyes were significantly smaller than the fellow eyes in unilateral ptosis children, which suggested that ptosis may lead to a delayed eyeball development and a hyperopic refractive power.

The development of astigmatism in pediatric ptosis may due to the band like pressure caused by ptotic eyelid on the cornea, which changes corneal shape contour [28,29]. However, altered cornea curvature had also been observed in form deprivation animal models and it might relate to the occurrence of astigmatism in ptosis patients [30,31,32], other than just eyelid compression and tightness on the cornea [33,34,35]. There was no significant difference in the risk of astigmatism among age groups. The result may support the theory that congenital ptosis might not cause significantly more severe anisometropia or astigmatism but might disturb the binocular balance by the disruption of fusion [36].

Previous research has documented that childhood ptosis is associated with increased risk of amblyopia [37,38,39]. The causal mechanism of functional deprivation amblyopia is the sustained reduction or lack of neural transmission of signals from the impaired eye to the lateral geniculate nucleus (LGN), leading to atrophy of the neural components [40,41,42]. Several large retrospective studies have shown that the main causes of amblyopia were strabismus and significant refractive error among patients with congenital ptosis [8,43]. In contrast, Griepentrog et al. have found that occlusion of the visual axis was the leading cause of amblyopia in congenital ptosis [18]. Furthermore, we found the prevalence of amblyopia had been increasing from 2002 to 2012 in children with and without ptosis. In line with previous studies, the amblyopia incidence generally increased over the past decades [44]. This is probably due to the increased detection of amblyopia and the elevated incidence of premature birth and survival, which has a significant association with amblyopia [45,46,47].

Some theories may explain our results of there being no statistically significant difference between the risk of amblyopia in early and late diagnosis groups. Synaptogenesis in the cortex develops from birth and stabilizes at about age 11 [48]. The process of pruning the initial overgrowth synapses that starts from 8–9 months after birth was thought to be a significant period of physiological and behavioral changes in visual function, which has a high relation with forming amblyopia. We speculate that the long-term process of synaptogenesis may be the reason for the risk of amblyopia not being significantly different between early and late onset age groups in childhood ptosis.

The strength of this study is the source of patients was a nationwide population-based database. The dataset used in this study is a subset of the NHIRD, which comprised half of all children in Taiwan from 2000 to 2013 and reduced selection bias. All participants were assigned a unique personal identification number and could be traced through the NHIRD during the study period. The use of a longitudinal study to monitor refractive errors and amblyopia development between cohorts with and without childhood ptosis was more effective than a medical chart review or cross-sectional study.

Nevertheless, some limitations should be noted. First, although the association between strabismus and ptosis has been well identified, it was not analyzed in this study. Second, several confounding variables, such as whether the patient received refractive correction or surgery, the timing of surgery for ptosis, reading habits, light exposure time, and family history, were not available from the administrative database. Finally, some asymptomatic or mildly symptomatic refractive errors patients may not have visited ophthalmologic clinics, which could lead to misclassification bias. However, most patients with refractive errors are included in the database because it is mandatory in Taiwan that kindergarten, elementary, and high school students should receive visual acuity examinations every 6 months using the logMAR chart. The students who exhibit a visual acuity less than 1.0 are asked to visit an ophthalmologist to confirm the results.

In conclusion, children with ptosis had higher risks of refractive errors and amblyopia. In addition, late ptosis diagnosis group (after age 3) had a higher risk of myopia, while there was no significant difference between the risk of hyperopia, amblyopia, and astigmatism in the early and late diagnosis group. These findings may have important implications in the prevention and management of children with ptosis.

## Figures and Tables

**Table 1:** Comparison of demographics and comorbidity between ptosis patients and controls.

	Ptosis		*p*-Value *
	No (N = 7187)	Yes (N = 1799)	
	*n* (%)	*n* (%)	
Age, years			0.95
≤3	4369 (60.79)	1094 (60.81)	
3–8	1821 (25.34)	451 (25.07)	
≥8	997 (13.87)	254 (14.12)	
Mean (SD)	3.77 (4.17)	3.72 (4.20)	0.6533
Sex			0.95
Girl	2863 (39.84)	718 (39.91)	
Boy	4324 (60.16)	1081 (60.09)	
Urbanization level ^†^			0.99
1 (highest)	2200 (30.66)	550 (30.62)	
2	2136 (29.77)	534 (29.73)	
3	1383 (19.28)	348 (19.38)	
4 (lowest)	1456 (20.29)	364 (20.27)	
Parental occupation			0.99
White collar	3560 (61.72)	890 (61.72)	
Blue collar	1196 (20.74)	299 (20.74)	
Others ^‡^	1012 (17.55)	253 (17.55)	

SD, standard deviation; * Chi-square test examined categorical data; *t*-test examined continuous. ^†^: The urbanization level was categorized by the population density of the residential area into 4 levels, with level 1 as the most urbanized and level 4 as the least urbanized. ^‡^ Other occupations included primarily retired, unemployed, or low-income populations.

**Table 2:** Incidence and adjusted hazard ratio of refractive errors and amblyopia for ptosis patients compared to controls.

	Ptosis			Non-Ptosis			Compared to Control	
Outcomes	Events	PY	Rate ^#^	Events	PY	Rate ^#^	Crude HR * (95% CI)	Adjusted HR ^†^ (95% CI)
Astigmatism	501	9362	53.51	389	45,985	8.46	6.22 (5.45, 7.10) ***	5.93 (5.16, 6.82) ***
Myopia	655	9115	71.86	953	43,970	21.67	3.48 (3.15, 3.85) ***	3.46 (3.13, 3.83) ***
Hyperopia	216	10,874	19.86	119	47,107	2.53	7.77 (6.21, 9.72) ***	7.60 (5.99, 9.63) ***
Amblyopia	309	10,339	29.89	97	47,256	2.05	14.24 (11.33, 17.88) ***	13.45 (10.60, 17.05) ***

PY, person-years; Rate ^#^, incidence rate per 1000 person-years; Crude HR *: relative hazard ratio; CI, confidence interval; Adjusted HR ^†^: adjusted hazard ratio controlling for age, sex, urbanization level and parental occupation; *** *p* < 0.001.

**Table 3:** Incidence and adjusted hazard ratio of refractive errors and amblyopia by sex for ptosis patients compared to controls.

Variables	Boy				Adjusted HR ^†^ (95% CI)	Girl				Adjusted HR ^†^ (95% CI)
	Ptosis					Ptosis				
	No		Yes			No		Yes		
	Event	Rate ^#^	Event	Rate ^#^		Event	Rate ^#^	Event	Rate ^#^	
Astigmatism	161	8.85	215	58.84	6.36 (5.12, 7.89) ***	228	8.21	286	50.1	5.67 (4.72, 6.81) ***
Myopia	384	22.05	258	69.97	3.22 (2.74, 3.78) ***	569	21.43	397	73.14	3.63 (3.19, 4.14) ***
Hyperopia	49	2.62	84	19.49	7.69 (5.29, 11.16) ***	70	2.46	132	20.11	7.53 (5.54, 10.24) ***
Amblyopia	44	2.35	123	29.88	11.11 (7.79, 15.84) ***	53	1.86	186	29.89	15.59 (11.31, 21.51) ***

Rate ^#^, incidence rate per 1000 person-years; CI, confidence interval; Adjusted HR ^†^: adjusted hazard ratio controlling for age, sex, urbanization level and parental occupation; *** *p* < 0.001.

**Table 4:** Incidence and adjusted hazard ratio of refractive errors and amblyopia by age for ptosis patients compared to controls.

Variables	Age ≤ 3				Adjusted HR ^†^ (95% CI)	Age > 3				Adjusted HR ^†^ (95% CI)
	Ptosis					Ptosis				
	No		Yes			No		Yes		
	Event	Rate ^#^	Event	Rate ^#^		Event	Rate ^#^	Event	Rate ^#^	
Astigmatism	161	8.85	215	58.84	6.08 (5.15, 7.17) ***	228	8.21	286	50.1	5.76 (4.46, 7.45) ***
Myopia	384	22.05	258	69.97	3.14 (2.75, 3.59) ***	569	21.43	397	73.14	3.90 (3.34, 4.56) ***
Hyperopia	49	2.62	84	19.49	7.03 (5.37, 9.22) ***	70	2.46	132	20.11	10.11 (6.15, 16.62) ***
Amblyopia	75	2.51	240	37.42	13.75 (10.46, 18.07) ***	22	1.27	69	17.58	13.21 (8.17, 21.37) ***

Rate ^#^, incidence rate per 1000 person-years; CI, confidence interval; Adjusted HR ^†^: adjusted hazard ratio controlling for age, sex, urbanization level and parental occupation; *** *p* < 0.001.

**Table 5:** Amblyopia prevalence of children in Taiwan (per 100).

	Non-Ptosis Children	Ptosis Children
2000	0.09	5.52
2001	0.15	6.17
2002	0.23	6.52
2003	0.31	7.45
2004	0.42	8.41
2005	0.53	9.08
2006	0.65	9.73
2007	0.79	10.63
2008	0.92	11.34
2009	1.07	11.92
2010	1.22	12.52
2011	1.38	12.93
2012	1.53	13.59
